# Supplementary material for: Above-room-temperature chiral skyrmion lattice and Dzyaloshinskii–Moriya interaction in a van der Waals ferromagnet Fe3−xGaTe2
Source: Nat Commun. 2024 May 25;15:4472. doi: 10.1038/s41467-024-48799-9 (PMC11127993; doi:10.1038/s41467-024-48799-9)
Supplement: Supplementary file 1 — Supplementary Information [file 41467_2024_48799_MOESM1_ESM.pdf]

Supplementary information

Above-room-temperature chiral skyrmion lattice and Dzyaloshinskii–Moriya interaction in a van der Waals ferromagnet $Fe_{3-x}GaTe_2$

Chenhui Zhang[1,5], Ze Jiang[2,5], Jiawei Jiang[3,5], Wa He[2], Junwei Zhang[2], Fanrui Hu[1], Shishun Zhao[1], Dongsheng Yang[1], Yakun Liu[1], Yong Peng[2,*], Hongxin Yang[3,4,*] & Hyunsoo Yang[1,*]

[1]Department of Electrical and Computer Engineering, National University of Singapore, Singapore 117576, Singapore

[2]School of Materials and Energy and Electron Microscopy Centre of Lanzhou University, Lanzhou University, Lanzhou 730000, China

[3]National Laboratory of Solid State Microstructures, School of Physics, Collaborative Innovation Center of Advanced Microstructures, Nanjing University, Nanjing 210093, China

[4]Center for Quantum Matter, School of Physics, Zhejiang University, Hangzhou 310058, China

[5]These authors contributed equally: Chenhui Zhang, Ze Jiang, Jiawei Jiang.

*e-mail: pengy@lzu.edu.cn; hongxin.yang@zju.edu.cn; eleyang@nus.edu.sg

This file includes:

Supplementary Note 1 | CVT-growth of $Fe_{3-x}GaTe_2$ single crystals

Supplementary Note 2 | Experimental estimation of DMI constant

Supplementary Note 3 | Details of DFT calculations

Supplementary Fig. 1 | Energy-dispersive X-ray spectrum of $Fe_{3-x}GaTe_2$

Supplementary Fig. 2 | XRD θ-2θ scan spectrum of $Fe_{3-x}GaTe_2$

Supplementary Fig. 3 | In-plane bulk isothermal magnetization of $Fe_{3-x}GaTe_2$

Supplementary Fig. 4 | Thickness determination of an $Fe_{3-x}GaTe_2$ flake

Supplementary Fig. 5 | L-TEM image of skyrmions and stripe domains under zero tilt angle

Supplementary Fig. 6 | Field-induced room-temperature magnetic skyrmions in $Fe_{3-x}GaTe_2$

Supplementary Fig. 7 | Field-cooled procedure to obtain obtain the skyrmion lattice

Supplementary Fig. 8 | Defocus- and tilt-angle-dependent L-TEM images of skyrmion lattice

Supplementary Fig. 9 | Estimation of average magnetic domain width

Supplementary Fig. 10 | Saturation magnetization fitted by the Bloch $T^{3/2}$ law

Supplementary Fig. 11 | Thickness determination of an hBN-covered $Fe_{3-x}GaTe_2$ flake

Supplementary Fig. 12 | EELS spectra of hBN-covered and uncovered $Fe_{3-x}GaTe_2$

Supplementary Fig. 13 | Defocus- and tilt-angle-dependent L-TEM images of labyrinth domains in hBN-protected $Fe_{3-x}GaTe_2$

Supplementary Fig. 14 | Field-dependent magnetic domain evolution in hBN-protected $Fe_{3-x}GaTe_2$

Supplementary Fig. 15 | Defocus- and tilt-angle-dependent L-TEM images of skyrmions in hBN-protected $Fe_{3-x}GaTe_2$

Supplementary Fig. 16 | Skyrmion size change in a positive magnetic field

Supplementary Fig. 17 | Displacement of Fe_{II} in the upper layer of the unit cell

Supplementary Fig. 18 | Comparison of the simulated and experimental HAADF-STEM images

Supplementary Fig. 19 | SAED patterns of $Fe_{3-x}GaTe_2$ lamellar samples

Supplementary Fig. 20 | Layer-resolved SOC energy difference ΔE_{SOC} associated with DMI distribution

Supplementary Fig. 21 | Skyrmion evolution in a negative magnetic field

Supplementary Fig. 22 | Sample thickness dependence of skyrmion size

Supplementary Table 1 | Summary of the SCXRD data and structure refinement

Supplementary Table 2 | Atomic coordinates of $Fe_{3-x}GaTe_2$

Supplementary Note 1 | CVT-growth of $Fe_{3-x}GaTe_2$ single crystals

$Fe_{3-x}GaTe_2$ single crystals have only been synthesized by using the flux method in previous studies[1-3]. Here we first report the growth of high-quality $Fe_{3-x}GaTe_2$ via a modified CVT method. We choose Fe (99.95%, Aladdin), GaTe (99.99%, Macklin), and Te (99.999%, Alfa Aesar) powders with a mole ratio of 3:1:1 as the starting materials, on the contrary to the elemental powders in the conventional CVT method. They are additionally mixed with a small amount of iodine (transport agent) and put into a quartz ampoule, which is subsequently evacuated and sealed. After one-week growth in a tube furnace with a temperature gradient of 750–700 °C, the ampoule is naturally cooled to room temperature. The chemical composition of the obtained crystals is determined to be $Fe_{2.85}GaTe_{2.03}$ via energy-dispersive X-ray spectroscopy (EDS; Supplementary Fig. 1). The CVT-grown crystals possess regular shapes and lustrous surfaces (inset of Supplementary Fig. 2), which are free of residual flux that usually remains on the flux-grown ones. In the X-ray diffraction (XRD) experiments, the bulk crystal is laid flat on the sample holder, and thus the (00*l*) peaks in the θ-2θ scan spectrum (Supplementary Fig. 2) imply that the *c* axis of the crystal is normal to the slab surface.

Supplementary Note 2 | Experimental estimation of DMI constant

Spin wave excitations determine the temperature dependence of magnetization. At low temperatures, the temperature-dependent saturation magnetization, $M_S(T)$, can be fitted with the Bloch $T^{3/2}$ law[4,5]:

$$\frac{M_S(T)}{M_S(0)} = 1 - BT^{3/2} \, , \qquad \text{(S1)}$$

where $M_S(0)$ is the saturation magnetization at 0 K and B is a spin-wave-related coefficient that is defined as[5]

$$B = \zeta(\tfrac{3}{2}) \frac{g\mu_B}{M_S(0)} (\frac{k_B}{4\pi D_{spin}(0)})^{3/2} \, . \qquad \text{(S2)}$$

Here, $\zeta(\frac{3}{2})$= 2.612 is the Riemann zeta function, g is the Landé g-factor, μ_B is the Bohr magneton, k_B is the Boltzmann constant, and $D_{spin}(0)$ is the spin-wave stiffness constant at 0 K.

Using the Bloch $T^{3/2}$ law fitting in Supplementary Fig. 10, we can obtain B= 5.04 × 10^{-5} $K^{-3/2}$ and $D_{spin}(0)$= 2.30 × 10^{-22} J nm^2. Thereafter, the zero-kelvin exchange stiffness constant $A(0)$ is calculated to be 1.97 pJ m^{-1} through the expression[5,6]:

$$A(0) = \frac{M_S(0)D_{spin}(0)}{2g\mu_B}. \quad \text{(S3)}$$

The temperature-dependent exchange stiffness constant, $A(T)$, can be further deduced through the following realtion[7]:

$$A(T) \cong A(0)\left[\frac{M_S(T)}{M_S(0)}\right]^{1+\gamma}, \quad \text{(S4)}$$

where the exponent γ is equal to 1 under the mean field regime[8]. Accordingly, the exchange stiffness constant A at 300 K is estimated to be 0.70 pJ m^{-1}.

For the effective magnetic anisotropy constant, it can be calculated using the area method[9]:

$$K_{eff} = \int_0^{M_S}[H_\perp(M) - H_\parallel(M)]\mathrm{d}M, \quad \text{(S5)}$$

where $H_\parallel$ and $H_\perp$ represent the out-of-plane and in-plane magnetic field, respectively. In practice, for hysteretic $M(H)$ loops, the hysteresis can be removed by averaging the two loop branches, and the K_{eff} can be calculated using the obtained anhysteretic curves[9-11]. Based on the isothermal magnetization results in Fig. 1b and Supplementary Fig. 3, the effective magnetic anisotropy constant at 300 K is determined to be K_{eff} = 0.30 MJ m^{-3}.

Note that the domain wall energy obtained by the phenomenological model using $\delta_w = \frac{wM_s^2}{4\pi\beta}$ in the main text is based on the L-TEM pattern acquired at 295 K (Supplementary Fig. 9). Whereas the exchange stiffness constant A and the effective magnetic anisotropy constant K_{eff} extracted here are at a slighly higher temperature of 300 K. We argue that this small temperature interval should not considerably change the micromagnetic parameters (one can see the nearly identical Hall resistance loops at 295 K and 300 K shown in Fig. 1c in the main text), thus the

domain wall energies at this two temperatures should be very close. Therefore, the DMI constant can be calculated as[12]

$$|D| = \frac{4\sqrt{AK_{\text{eff}}} - \delta_w}{\pi}, \tag{S6}$$

where δ_w= 0.22 mJ m^{-2} is obtained by the phenomenological model (see main text) and $4\sqrt{AK_{\text{eff}}}$= 1.83 mJ m^{-2} is extracted via the micromagnetic analysis. Finally, $|D|$= 0.51 mJ m^{-2} is obtained.

It is worth noting that the accurate and precise determination of DMI constant is difficult, and thus the value obtained here should be considered as an estimation. On the one hand, the effective magnetic anisotropy in the bulk and exfoliated samples may show discrepancy, i.e., K_{eff} can be thickness dependent. Considering the L-TEM sample used to extract the domain wall energy is quite thick (~179 nm), we suppose the magnetic properties of the bulk and this exfoliated flake are similar. Therefore, this K_{eff} discrepancy is supposed to be small. On the other hand, the values of coefficients, including β and γ, used in the calculations should be regard as reasonable estimations. The accurate determination of these coefficients in $Fe_{3-x}GaTe_2$ is difficult and beyond the scope of this study. For instance, in general cases, the magnetic critical behavior of itinerant ferromagnes may diverge from the mean field model. The critical exponent γ is supposed to be in the range from 0.7 to 4/3[7,13], thus the error caused by γ can be further estimated. Accordingly, we estimate that $|D|$ can be in the range of 0.46 – 0.56 mJ m^{-2}.

Supplementary Note 3 | Details of DFT calculations

The first-principles calculations are performed using the Vienna *ab initio* simulation package (VASP) with local density approximation for the exchange and correlation function[14-17]. Kohn-Sham single-particle wave functions are expanded in the plane wave basis set with a kinetic energy cutoff at 600 eV. The energy and force convergence criteria are 10^{-7} eV and 10^{-3} eV/Å,

respectively. Long-range correlation is included in evaluating vdW interaction by the optB86b method[18]. The standard $P6_3/mmc$ Fe_3GaTe_2 is fully relaxed as the initial structure, then the experimentally determined Fe_{II} displacement is imposed to get the calculation model. The crystal symmetry of the models is determined and analyzed by the commonly used "symmetry finder" and "Bilbao Crystallographic Server" tools. The microscopic and micromagnetic DMI parameters (*d* and *D*) are calculated using the real-space spin spiral method[19], in which a 4×1 supercell is used. The Brillouin zone is sampled by a Monkhorst-Pack 4×16×3 k-point grid. Such a method has been successfully implemented in both bulk materials and interfaces[20,21].

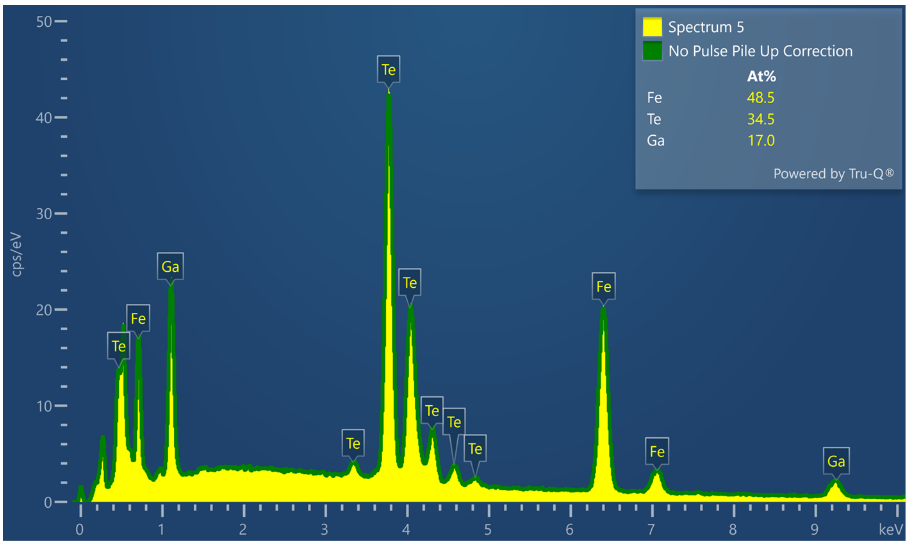

Supplementary Fig. 1 | Energy-dispersive X-ray spectrum of $Fe_{3-x}GaTe_2$. An atomic percentage ratio of Fe:Ga:Te = 48.5:17.0:34.5 is acquired, which leads to a chemical composition of $Fe_{2.85}GaTe_{2.03}$.

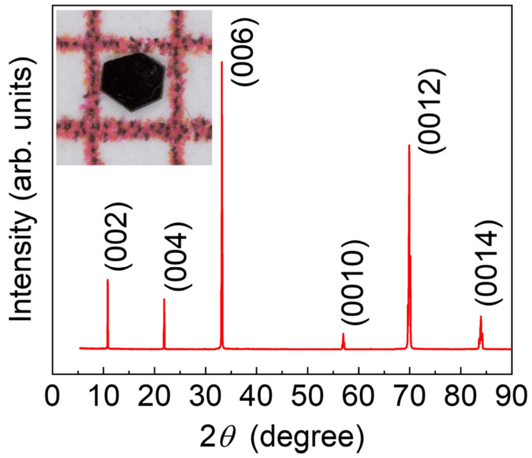

Supplementary Fig. 2 | XRD θ-2θ scan spectrum of $Fe_{3-x}GaTe_2$. The inset is the optical image of an as-grown $Fe_{3-x}GaTe_2$ crystal, in which the grid size is 1 mm.

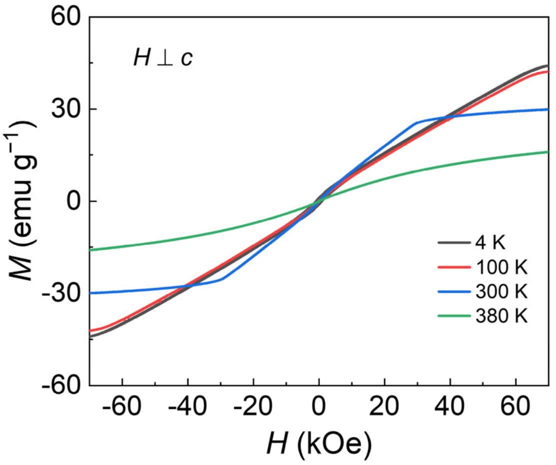

Supplementary Fig. 3 | In-plane bulk isothermal magnetization of $Fe_{3-x}GaTe_2$.

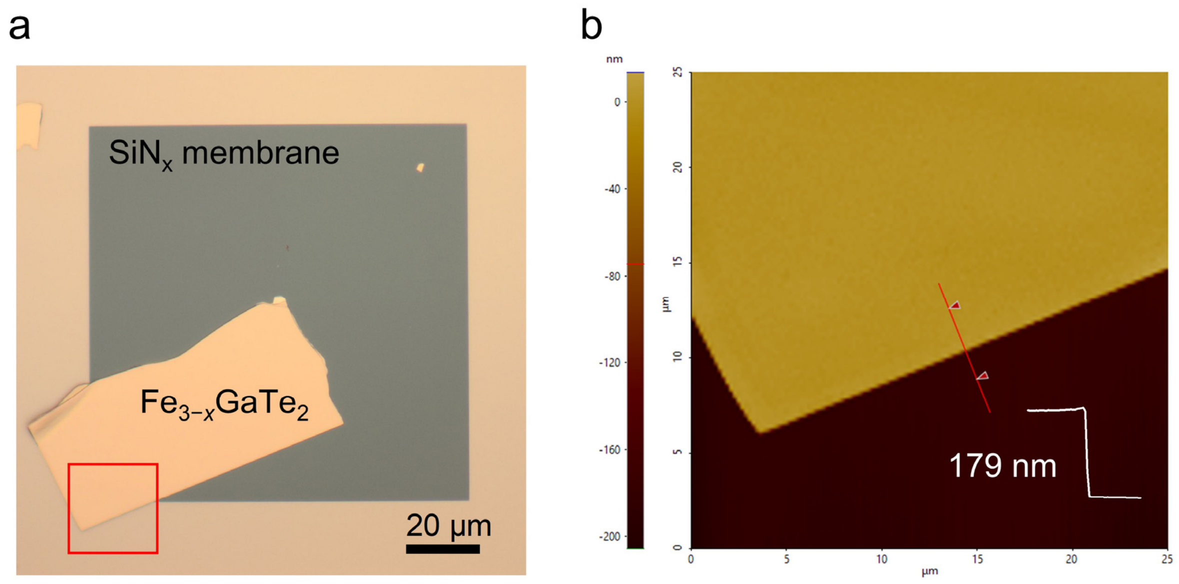

Supplementary Fig. 4 | Thickness determination of an $Fe_{3-x}GaTe_2$ flake. a Optical image of an $Fe_{3-x}GaTe_2$ flake on a silicon nitride TEM grid. **b** AFM image of the red square region in **a**. The white curve is the AFM height profile of the cross-section marked by the red line.

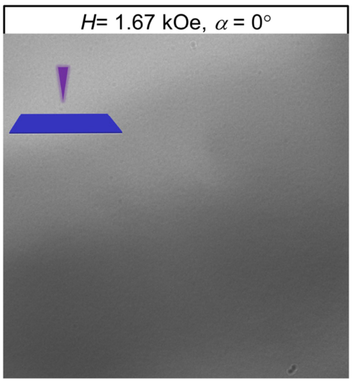

Supplementary Fig. 5 | L-TEM image of skyrmions and stripe domains under zero tilt angle. The defocus value is −3 mm and the temperature is 295 K.

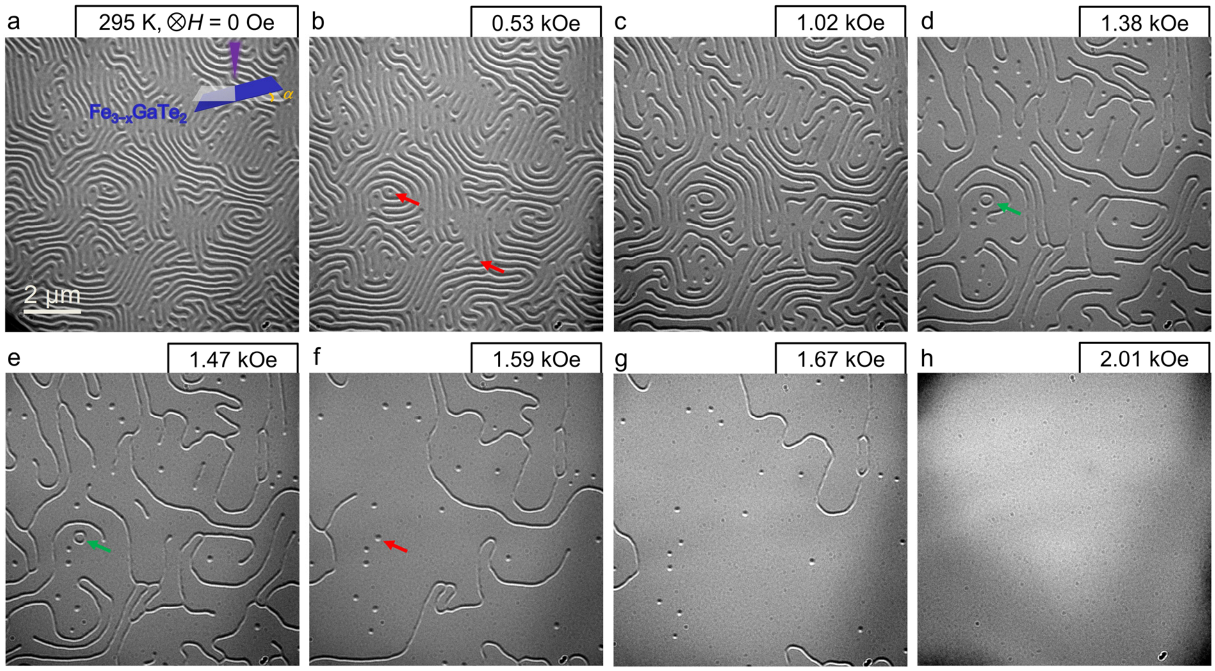

Supplementary Fig. 6 | Field-induced room-temperature magnetic skyrmions in $Fe_{3-x}GaTe_2$. a–h Evolution of magnetic domains in a 179 nm-thick $Fe_{3-x}GaTe_2$ flake under an out-of-plane magnetic field. The sample is ZFC from 370 to 295 K before imaging. The defocus value is −3 mm and the tilt angle α is 11°. Several Néel-type magnetic skyrmions and skyrmioniums are pointed out by the red and green arrows, respectively.

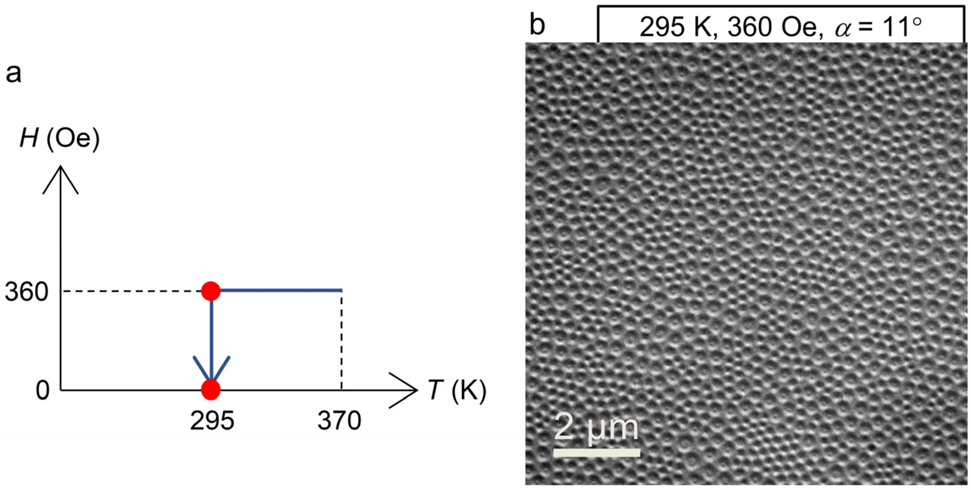

Supplementary Fig. 7 | Field-cooled procedure to obtain the skyrmion lattice. a Schematic diagram of the FC procedure. **b** L-TEM image obtained at 360 Oe after the sample is FC from 370 to 295 K. The defocus value is −3 mm and the tilt angle is 11°.

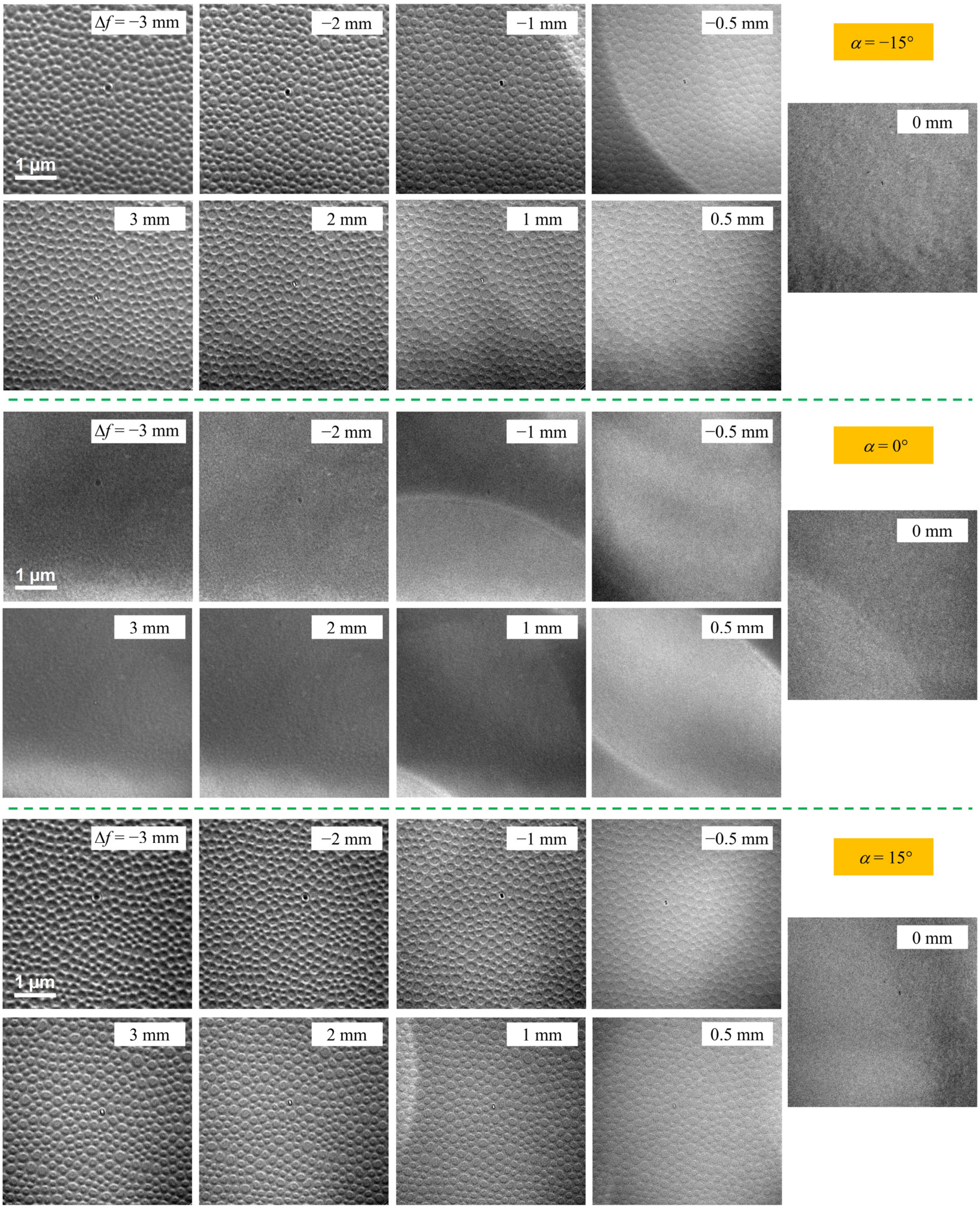

Supplementary Fig. 8 | Defocus- and tilt-angle-dependent L-TEM images of skyrmion lattice. Before imaging, the sample is FC from 370 to 295 K under an out-of-plane magnetic field of 360 Oe, and subsequently the field is removed.

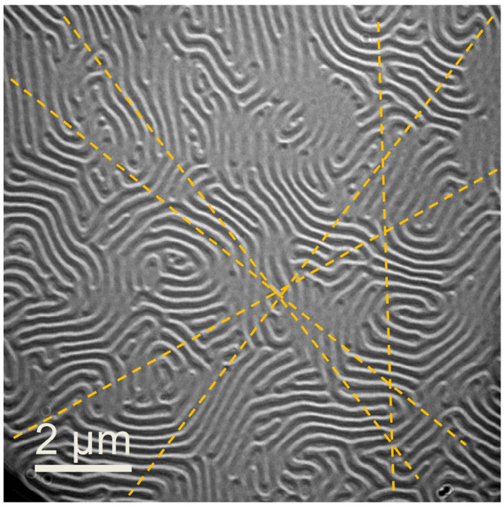

Supplementary Fig. 9 | Estimation of average magnetic domain width. The domain pattern is reproduced from Fig. 1d in the main text. The orange dashed lines indicate the five test lines which are randomly chosen.

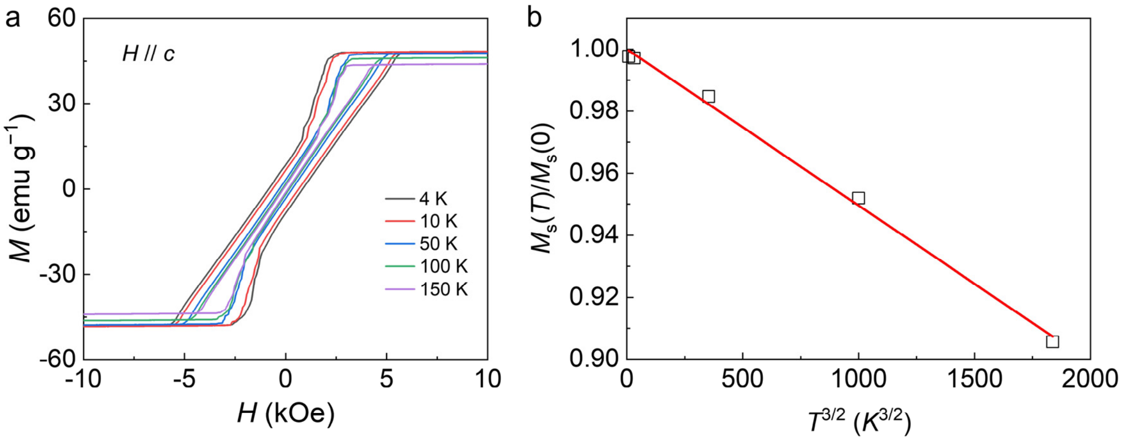

Supplementary Fig. 10 | Saturation magnetization fitted by the Bloch $T^{3/2}$ law. a Out-of-plane bulk isothermal magnetization at low temperatures. **b** The Bloch $T^{3/2}$ law fitting.

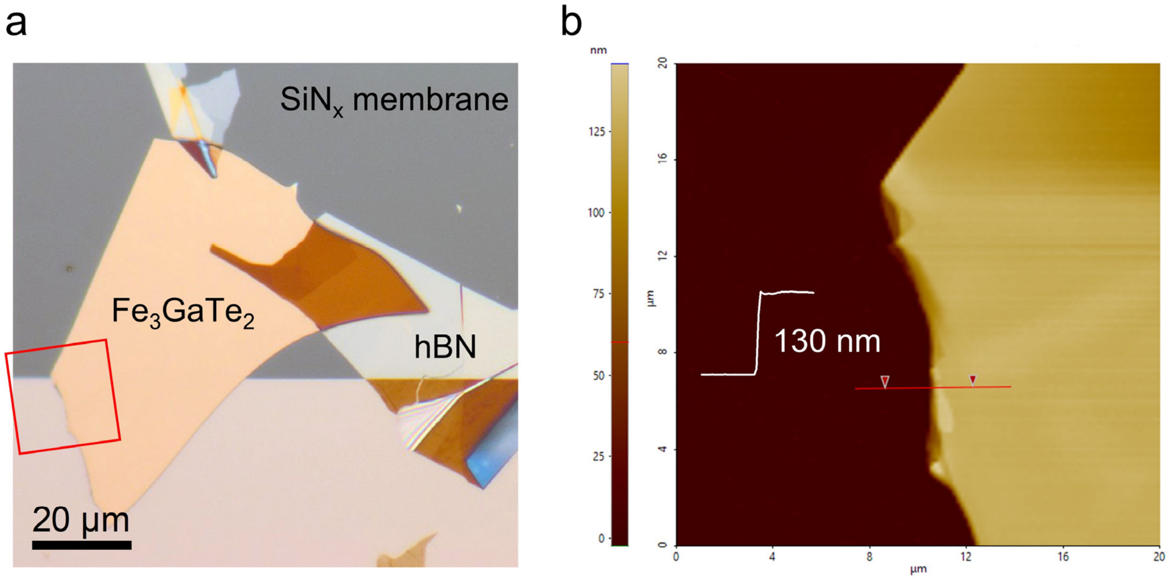

Supplementary Fig. 11 | Thickness determination of an hBN-covered $Fe_{3-x}GaTe_2$ flake. a Optical image of an hBN-covered $Fe_{3-x}GaTe_2$ flake on a silicon nitride TEM grid. **b** AFM image of the red square region in **a**. The white curve is the AFM height profile of the cross-section marked by the red line.

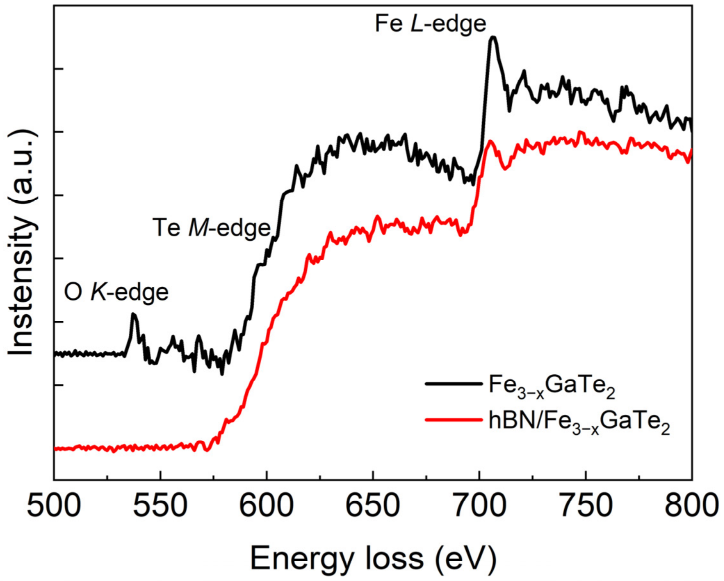

Supplementary Fig. 12 | EELS spectra of hBN-covered and uncovered $Fe_{3-x}GaTe_2$. The oxygen *K* edge is observed in uncovered regions, whereas it is undetected in hBN-covered regions.

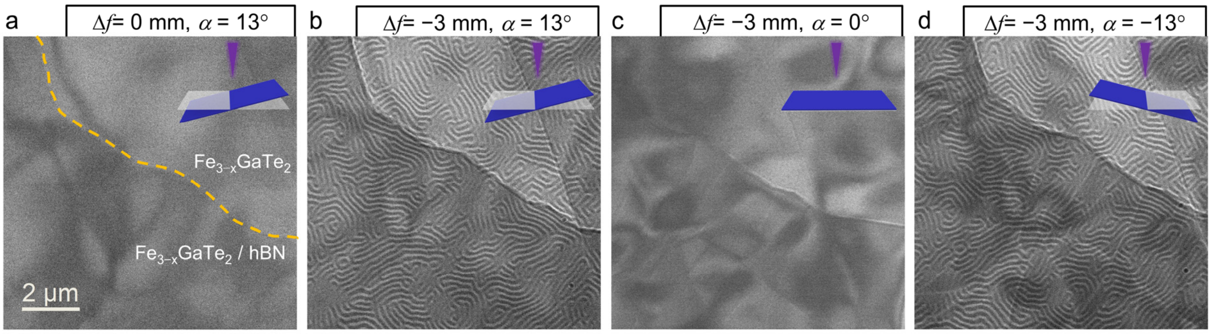

Supplementary Fig. 13 | Defocus- and tilt-angle-dependent L-TEM images of labyrinth domains in hBN-protected $Fe_{3-x}GaTe_2$. **a** In-focus (Δf = 0 mm) L-TEM image of a 130 nm-thick $Fe_{3-x}GaTe_2$ flake which is partially covered by hBN. **b–d** Under-focus (Δf = −3 mm) L-TEM images taken under different tilt angles. The sample is ZFC from 370 to 320 K before imaging.

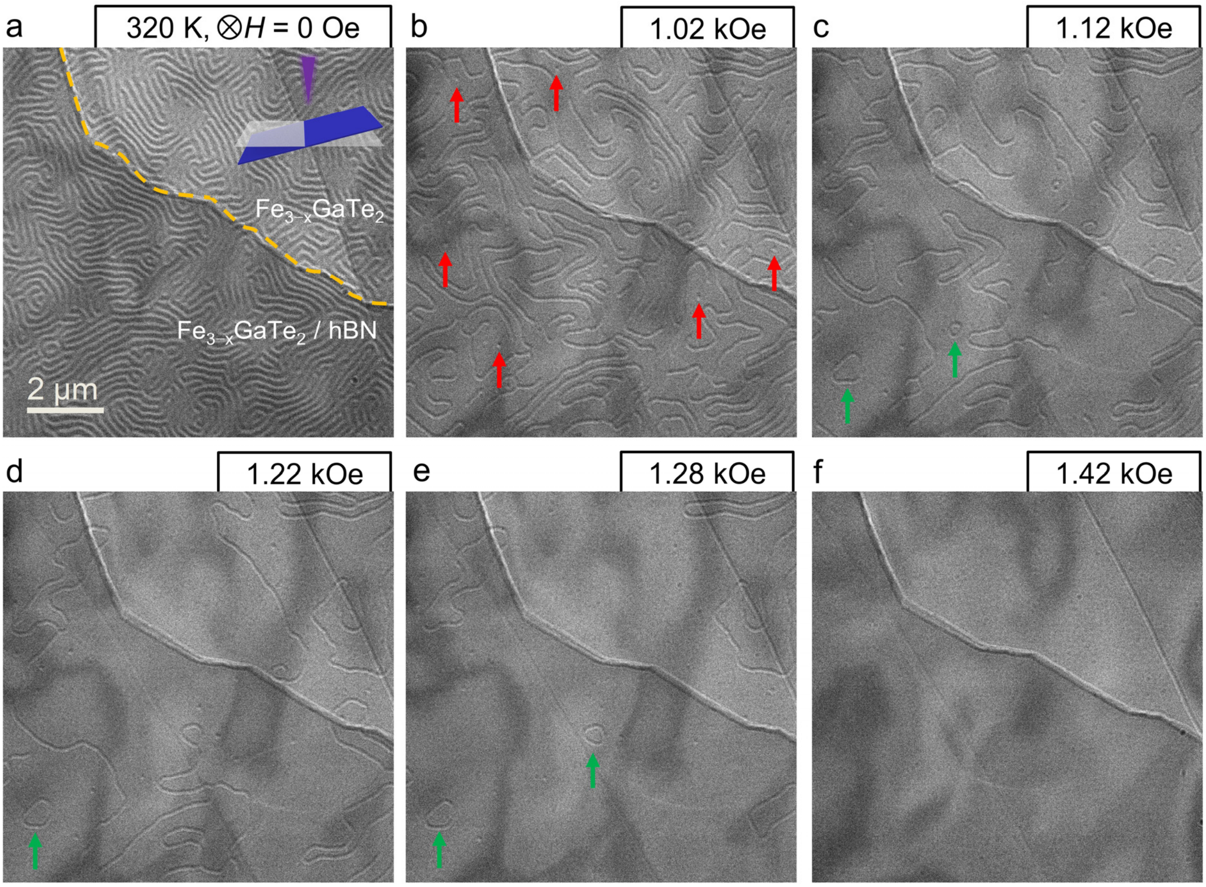

Supplementary Fig. 14 | Field-dependent magnetic domain evolution in hBN-protected $Fe_{3-x}GaTe_2$. The sample is ZFC from 370 to 320 K before imaging. The defocus value is −3 mm and the tilt angle is 13°. Several Néel-type magnetic skyrmions and skyrmioniums are pointed out by the red and green arrows, respectively.

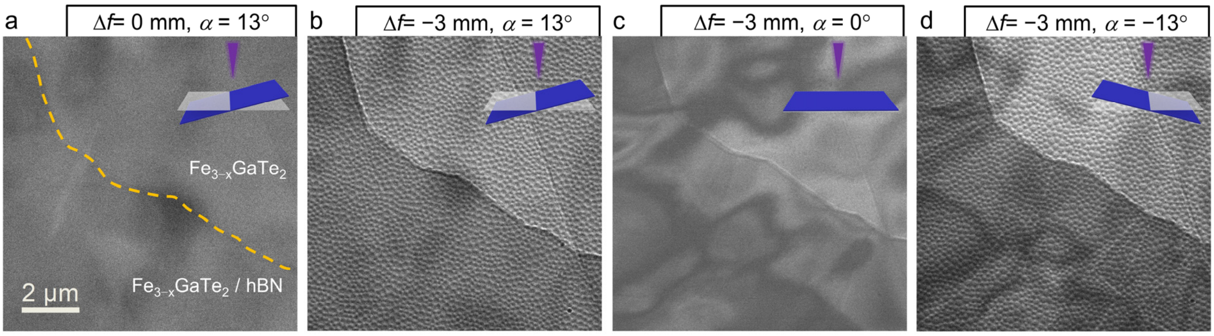

Supplementary Fig. 15 | Defocus- and tilt-angle-dependent L-TEM images of skyrmions in hBN-protected $Fe_{3-x}GaTe_2$. a In-focus (Δf = 0 mm) L-TEM inage of a 130 nm-thick $Fe_{3-x}GaTe_2$ flake which is partially covered by hBN. **b–d** Under-focus ($\Delta f = -3$ mm) L-TEM images taken under different tilt angles. Before imaging, the sample is FC from 370 to 300 K under an out-of-plane magnetic field of 360 Oe, and subsequently the field is removed.

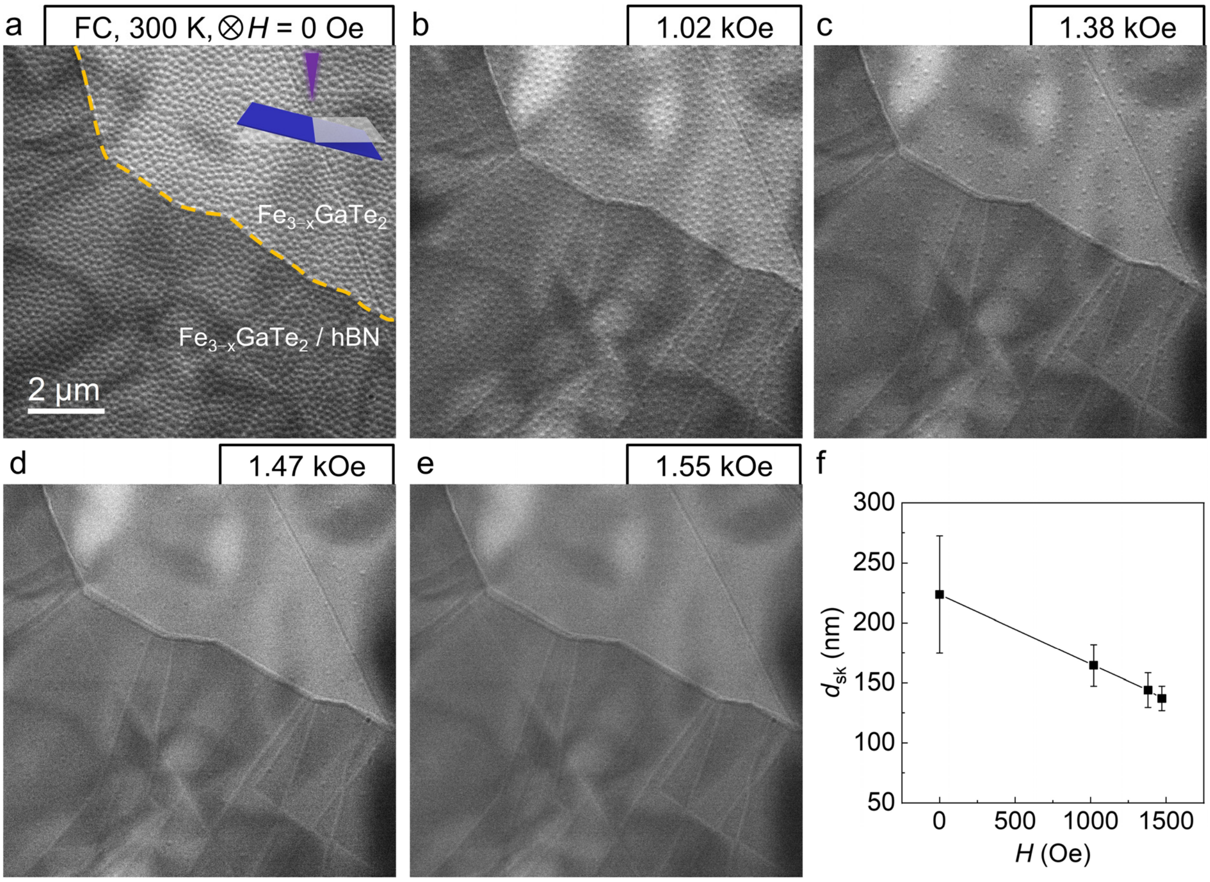

Supplementary Fig. 16 | Skyrmion size change in a positive magnetic field. Before imaging, the sample is FC from 370 to 300 K under an out-of-plane magnetic field of 360 Oe, and subsequently the field is removed. Then the field gradually increases from **a** to **e**. In **f**, d_{sk} represents the diameter of the skyrmion. The defocus value is −3 mm and the tilt angle is −13°.

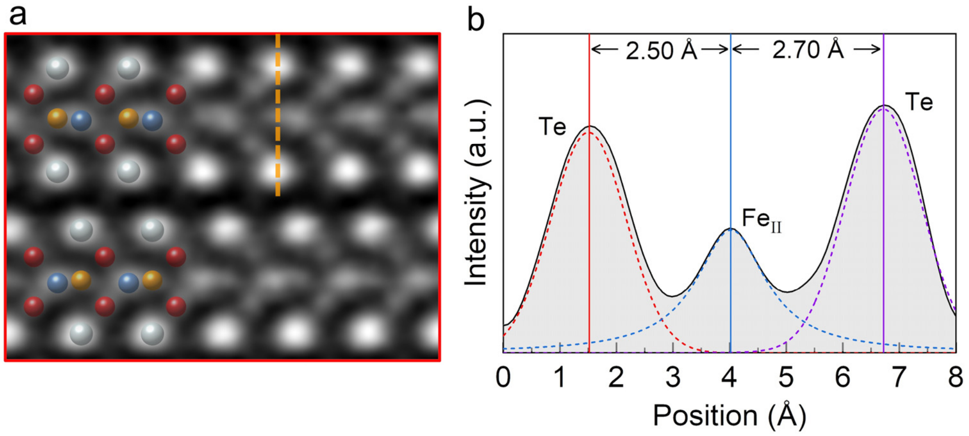

Supplementary Fig. 17 | Displacement of Fe$_{II}$ in the upper layer of the unit cell. a The same HAADF-STEM image in Fig. 4b in the main text. **b** Intensity profile of the orange dashed lines in **a**. The profile is fitted by the Voigt function (dashed curves). The peak positions are indicated by the vertical lines.

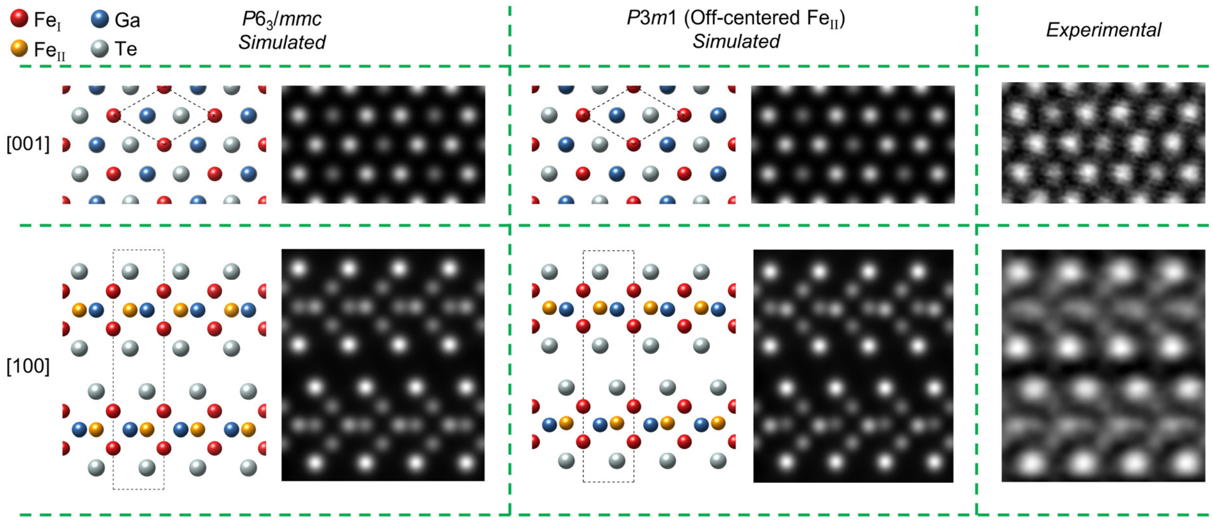

Supplementary Fig. 18 | Comparison of the simulated and experimental HAADF-STEM images. The left, middle, and right column shows the simulated $P6_3/mmc$, simulated $P3m1$, and experimental images, respectively. The black dashed lines denote the unit cell. The simulated HAADF-STEM images are acquired by using the Dr. Probe software[22].

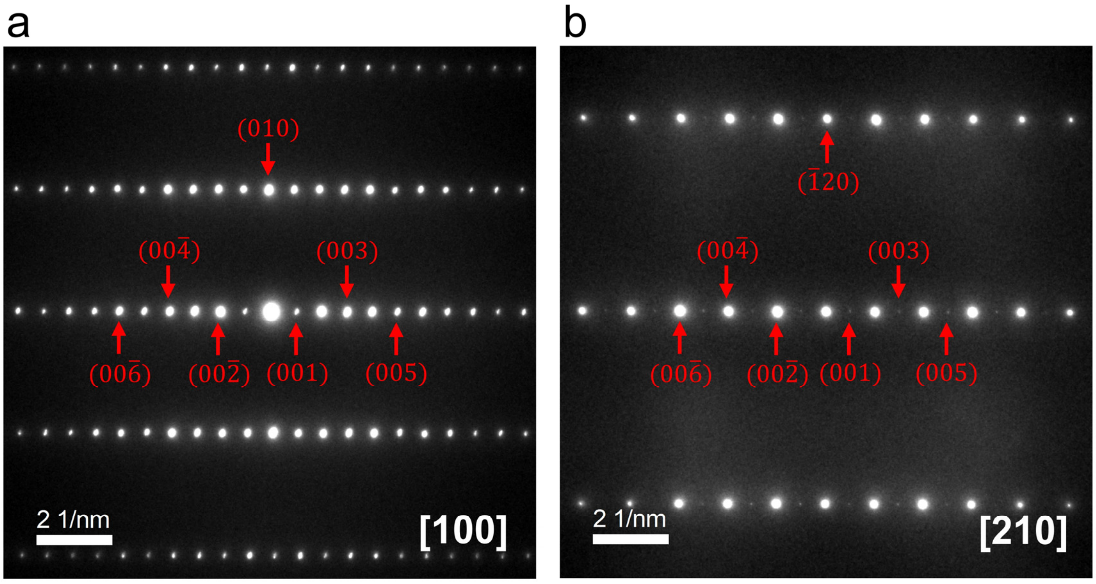

Supplementary Fig. 19 | SAED patterns of $Fe_{3-x}GaTe_2$ lamellar samples. The patterns are taken along the [100] (**a**) and [210] (**b**) zone axes.

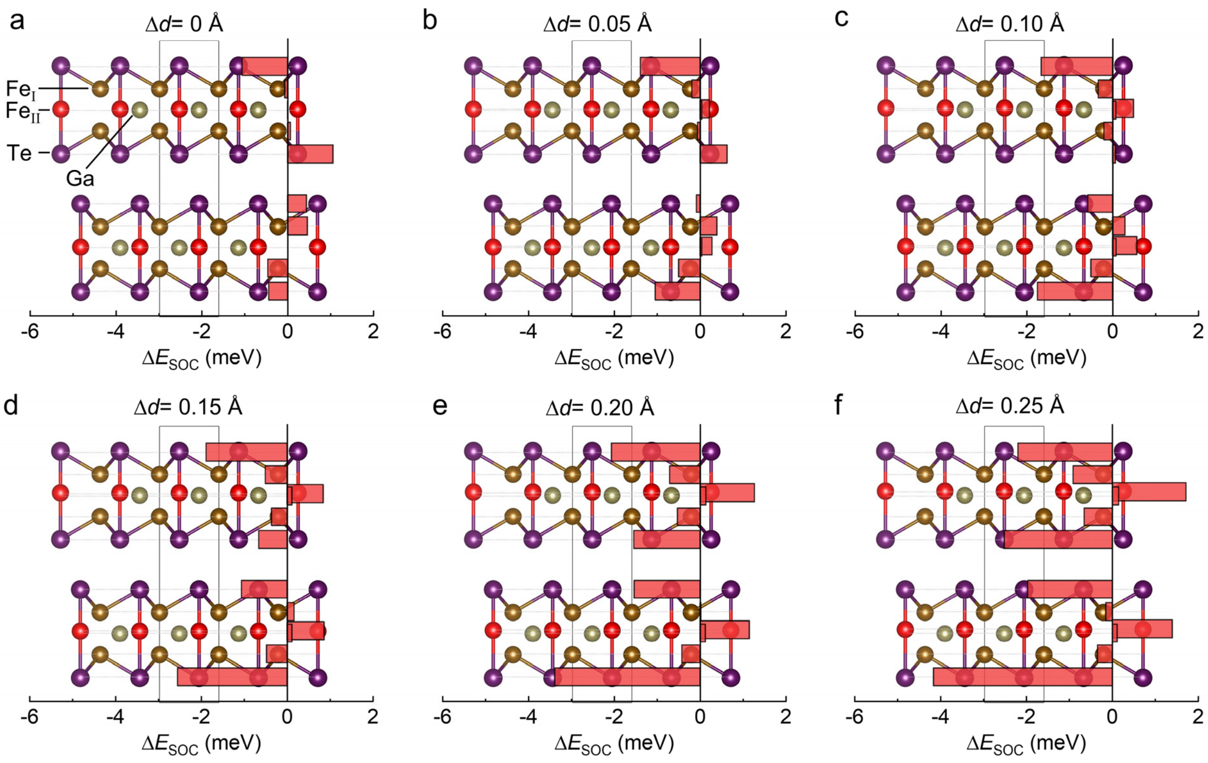

Supplementary Fig. 20 | Layer-resolved SOC energy difference ΔE_{SOC} associated with DMI distribution. For simplicity, the vertical displacements of Fe_{II} atoms (Δd) in both upper and lower layers are set to the same value of 0 Å (**a**), 0.05 Å (**b**), 0.10 (**c**) Å, 0.15 Å (**d**), 0.20 Å (**e**), and 0.25 Å (**f**).

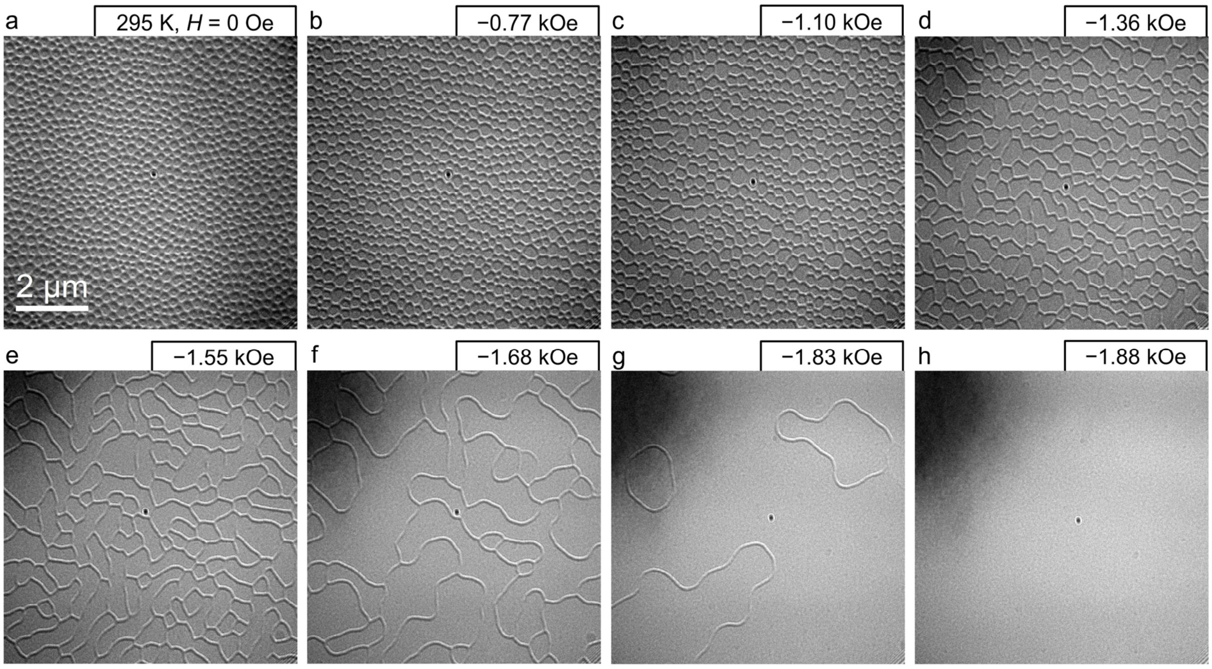

Supplementary Fig. 21 | Skyrmion evolution in a negative magnetic field. Before imaging, the sample is FC from 370 to 295 K under an out-of-plane magnetic field of 360 Oe, and subsequently the field is removed. Then an opposite magnetic field gradually increases from 0 to 1.88 kOe. The defocus value is −2 mm and the tilt angle is 15°.

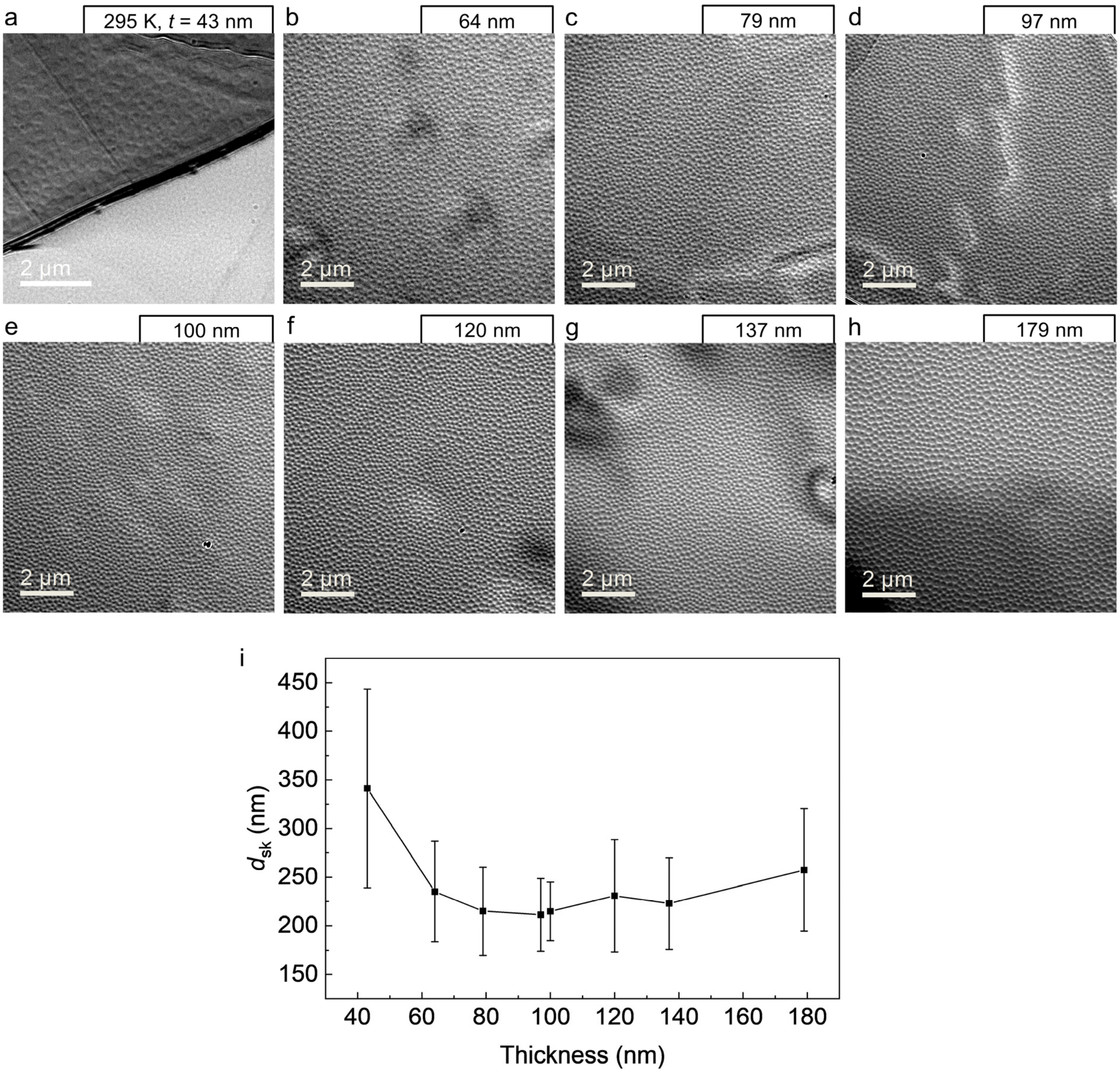

Supplementary Fig. 22 | Sample thickness dependence of skyrmion size. Before imaging, the sample is FC from 370 to 295 K under an out-of-plane magnetic field of 360 Oe, and subsequently the field is removed. The defocus value is −3 mm and the tilt angle is 11°. d_{sk} represents the diameter of the skyrmion.

Supplementary Table 1 | Summary of the SCXRD data and structure refinement.

Chemical formula	$Fe_{2.78}GaTe_2$
Formula weight (g mol^{-1})	480.34
Temperature (K)	273(2)
Crystal system	trigonal
Space group	*P*3*m*1 (No. 156)
a (Å)	4.0826(3)
b (Å)	4.0826(3)
c (Å)	16.1465(16)
α (°)	90
β (°)	90
γ (°)	120
Volume (Å^3)	233.07(4)
Z	2
Density ρ_{calc} (g cm^{-3})	6.845
Absorption coefficient μ (mm^{-1})	26.286
F(000)	415
Radiation	Mo Kα (λ = 0.71073 Å)
θ range for data collection (°)	2.52 to 28.27
Index ranges	$-5 \leq h \leq 5$, $-5 \leq k \leq 5$, $-21 \leq l \leq 21$
Reflections collected	2369
Independent reflections	558 [R_{int} = 0.0467, R_{sigma} = 0.0433]
Completeness	98.9%
Structure solution technique	direct methods
Structure solution program	SHELXT
Refinement method	Full-matrix least-squares on F^2
Refinement program	SHELXT
Function minimized	$\Sigma\, w(F_o^2 - F_c^2)^2$
Data/restraints/parameters	558/1/38
Goodness-of-fit on F^2	1.116
Final R indexes [$I > 2\sigma(I)$]	R_1 = 0.0600, wR_2 = 0.1667
Final R indexes [all data]	R_1 = 0.0624, wR_2 = 0.1720
Weighting scheme	$w=1/[\sigma^2(F_o^2) + (0.1259P)^2 + 0.8545P]$ where $P= (F_o^2 + 2F_c^2)/3$
Absolute structure parameter	0.4(2)
Largest diff. peak/hole (*e*Å^{-3})	5.970/−3.541
R.M.S. deviation from mean (*e*Å^{-3})	0.607

Supplementary Table 2 | Atomic coordinates of $Fe_{3-x}GaTe_2$. The equivalent isotropic atomic displacement parameter U_{eq} is defined as 1/3 of the trace of the orthogonalised U_{ij} tensor.

Atom	*x*	*y*	*z*	U_{eq} (Å^2)	Occupancy
Fe1	0.333333	0.666667	0.2546(6)	0.015(2)	0.8509
Fe2	0.0	0.0	0.3270(6)	0.0132(19)	0.8912
Fe3	0.0	0.0	0.8280(6)	0.0164(19)	1
Fe4	0.0	0.0	0.1741(8)	0.026(3)	1
Fe5	0.0	0.0	0.6748(8)	0.018(3)	0.9032
Fe6	0.666667	0.333333	0.7543(6)	0.0126(16)	0.92
Ga1	0.333333	0.666667	0.7476(7)	0.0294(19)	1
Ga2	0.666667	0.333333	0.2468(7)	0.0304(18)	1
Te1	0.333333	0.666667	0.40883(19)	0.0190(8)	1
Te2	0.333333	0.666667	0.0909(2)	0.0240(10)	1
Te3	0.666667	0.333333	0.90860(19)	0.0168(8)	1
Te4	0.666667	0.333333	0.5907(2)	0.0229(10)	1

Supplementary References

1. Zhang, G. et al. Above-room-temperature strong intrinsic ferromagnetism in 2D van der Waals Fe_3GaTe_2 with large perpendicular magnetic anisotropy. *Nat. Commun.* **13**, 5067 (2022).
2. Pan, H. et al. Room-Temperature Lateral Spin Valve in Graphene/Fe_3GaTe_2 van der Waals Heterostructures. *ACS Mater. Lett.* **5**, 2226-2232 (2023).
3. Ahn, H. B. et al. Giant coercivity enhancement in a room-temperature van der Waals magnet through substitutional metal-doping. *Nanoscale* **15**, 11290-11298 (2023).
4. Umetsu, R. Y., Okubo, A., Fujita, A., Kanomata, T., Ishida, K. & Kainuma, R. Spin Wave-Stiffness Constants of Half-Metallic Ferromagnets Co_2YZ (Y= Cr, Mn, and Fe, Z= Ga, Al, and Si) Heusler Alloys. *IEEE Trans. Magn.* **47**, 2451-2454 (2011).
5. Vaz, C. A. F., Bland, J. A. C. & Lauhoff, G. Magnetism in ultrathin film structures. *Rep. Prog. Phys.* **71**, 056501 (2008).
6. Šipr, O., Mankovsky, S. & Ebert, H. Spin wave stiffness and exchange stiffness of doped permalloy via ab initio calculations. *Phys. Rev. B* **100**, 024435 (2019).
7. Nembach, H. T., Shaw, J. M., Weiler, M., Jué, E. & Silva, T. J. Linear relation between Heisenberg exchange and interfacial Dzyaloshinskii–Moriya interaction in metal films. *Nat. Phys.* **11**, 825-829 (2015).
8. Kaul, S. Static critical phenomena in ferromagnets with quenched disorder. *J. Magn. Magn. Mater.* **53**, 5-53 (1985).
9. Johnson, M. T., Bloemen, P. J. H., Broeder, F. J. A. d. & Vries, J. J. d. Magnetic anisotropy in metallic multilayers. *Rep. Prog. Phys.* **59**, 1409-1458 (1996).
10. Bloemen, P. J. H. & de Jonge, W. J. M. Magnetic anisotropy of Co/Ni/Co/Pt multilayers. *J. Magn. Magn. Mater.* **116**, L1-L6 (1992).

11. Srinivasan, K., Chen, Y., Cestarollo, L., Dare, D. K., Wright, J. G. & El-Ghazaly, A. Engineering large perpendicular magnetic anisotropy in amorphous ferrimagnetic gadolinium cobalt alloys. *J. Mater. Chem. C* **11**, 4820-4829 (2023).

12. Heide, M., Bihlmayer, G. & Blügel, S. Dzyaloshinskii-Moriya interaction accounting for the orientation of magnetic domains in ultrathin films: Fe/W(110). *Phys. Rev. B* **78**, 140403(R) (2008).

13. Kim, S. et al. Correlation of the Dzyaloshinskii-Moriya interaction with Heisenberg exchange and orbital asphericity. *Nat. Commun.* **9**, 1648 (2018).

14. Kresse, G. & Joubert, D. From ultrasoft pseudopotentials to the projector augmented-wave method. *Phys. Rev. B* **59**, 1758-1775 (1999).

15. Kresse, G. & Furthmüller, J. Efficient iterative schemes for *ab initio* total-energy calculations using a plane-wave basis set. *Phys. Rev. B* **54**, 11169-11186 (1996).

16. Perdew, J. P. & Zunger, A. Self-interaction correction to density-functional approximations for many-electron systems. *Phys. Rev. B* **23**, 5048-5079 (1981).

17. Ceperley, D. M. & Alder, B. J. Ground State of the Electron Gas by a Stochastic Method. *Phys. Rev. Lett.* **45**, 566-569 (1980).

18. Klimeš, J., Bowler, D. R. & Michaelides, A. Van der Waals density functionals applied to solids. *Phys. Rev. B* **83**, 195131 (2011).

19. Yang, H., Thiaville, A., Rohart, S., Fert, A. & Chshiev, M. Anatomy of Dzyaloshinskii-Moriya Interaction at Co/Pt Interfaces. *Phys. Rev. Lett.* **115**, 267210 (2015).

20. Yang, H. et al. Significant Dzyaloshinskii-Moriya interaction at graphene-ferromagnet interfaces due to the Rashba effect. *Nat. Mater.* **17**, 605-609 (2018).

21. Yang, H., Liang, J. & Cui, Q. First-principles calculations for Dzyaloshinskii–Moriya interaction. *Nat. Rev. Phys.* **5**, 43-61 (2023).

22. Barthel, J. Dr. Probe: A software for high-resolution STEM image simulation. *Ultramicroscopy* **193**, 1-11 (2018).
